# DON’T STOP BELIEVING – MAKING AGE MATTER

**DOI:** 10.1093/geroni/igad104.1106

**Published:** 2023-12-21

**Authors:** Joann Montepare

**Affiliations:** Lasell University, Newton, Massachusetts, United States

## Abstract

As educators and scholars in the field of aging, many of us have seen how ageism operates in nuanced and powerful ways on many fronts and coming from different directions. In this presentation I will use examples from my personal experiences in higher education to describe how age biases can rear their heads and how they have guided me to respond. Beginning with my experiences as a junior faculty member in the 1980s when lifespan developmental approach was reshaping the traditional view of aging, I will share how attitudes about “age doesn’t matter” prevailed and warranted brave and creative responses. Reshaping courses from the inside out to integrate aging became my mission and passion. Whether it a traditional developmental psychology class, a core social psychology class, or a specialized class on face perception – aging could be shown to matter. Fast forward 40 years and much as come to fruition, as seen in the response to the Age-Friendly University (AFU) initiative that I have been helping to build with AGHE leaders, GSA colleagues, and AFU partners. However, there is much more work to be done as we see ourselves being the older adult in our courses about whom we have discussed and the older faculty member in an organizational structure built around people of a certain age. However, we know that if we “Don’t stop believin’, hold on to that feelin” we can continue to pave the way for age inclusivity in even more impactful ways.

